# Current status, challenges, and opportunities for sustainable crop production in Xinjiang

**DOI:** 10.1016/j.isci.2025.112114

**Published:** 2025-02-27

**Authors:** Xiaofeng Zhao, Annah Lake Zhu, Xiaohuang Liu, Hongyu Li, Haoyang Tao, Xiaoxia Guo, Jiufen Liu

**Affiliations:** 1 Comprehensive Survey Command Center for Natural Resources, China Geological Survey, Beijing 100055, China; 2 School of Earth Science and Resources, China University of Geosciences (Beijing), Beijing 100083, China; 3 Key Laboratory of Coupling Process and Effect of Natural Resources Elements, Beijing 100055, China; 4 Environmental Policy Group, Wageningen University and Research, Hollandseweg 1, 6706 KN Wageningen, the Netherlands; 5 College of Resources and Environmental Sciences, China Agricultural University, Beijing 100193, China; 6 College of Forestry, Henan Agricultural University, Zhengzhou 450002, China; 7 Technology Innovation Center for Analysis and Detection of the Elemental Speciation and Emerging Contaminants, China Geological Survey, Kunming 650100, China

**Keywords:** Environmental management, Agricultural science, Relation between agriculture and environment

## Abstract

Xinjiang is China’s largest cotton and grape production base, but faces challenges like water scarcity, overuse of fertilizer and mulching films, and low resource use efficiency. This review summarizes crop production in Xinjiang from 2000 to 2020, including geography, climate, sowing structure, yield variations, and barriers to sustainable management. Based on our review, we identify potential technical solutions (i.e., precise irrigation and fertilization, organic fertilizer substitution, mulching film management, and suitability evaluation of arable lands) and policy support (i.e., national plans, laws, and financial support) required to overcome these barriers. Overall, the sustainable transformation of crop production in Xinjiang is a challenging yet promising endeavor. Implementing judicious to make full use of water, heat, and nutrition resources have great potential for achieving the synergies between high yield, resource utilization efficiency, and environmental benefits. The findings also have global implications for other regions dominated by arid and oasis agriculture.

## Graphical abstract

**Figure:**
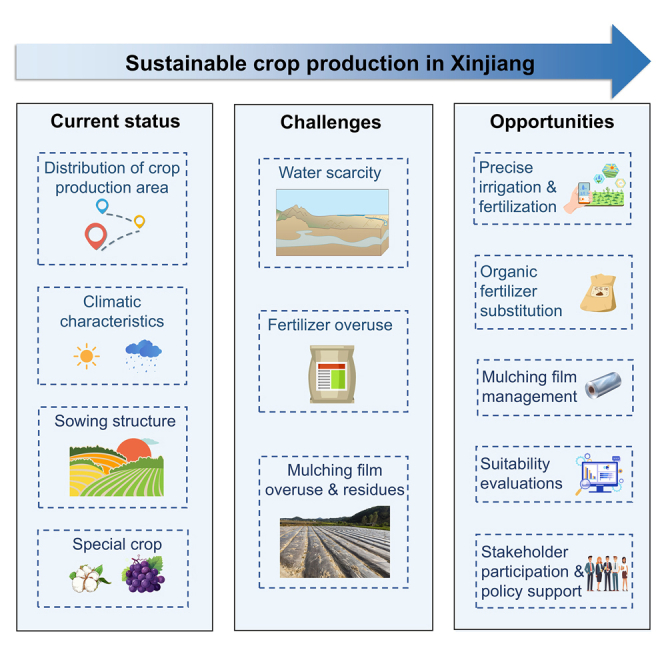

## Introduction

Xinjiang Uygur Autonomous Region (Xinjiang) is located in the northwestern China and stands as the largest province-level division within the country, covering an expansive area of 1.66 × 10^6^ km^2^. Xinjiang is characterized by its vast territory and sparse population, accounting for 17.3% of China’s land area but only 1.8% of its population.^1^ Xinjiang has a vast territory and large areas of arable land, making it highly suitable for mechanized operations, which reduce labor time and improve labor productivity. In 2024, the comprehensive mechanization rates for the cultivation, sowing, and harvesting of wheat, corn, and cotton in Xinjiang reached 99.5%, 95.5%, and 97%, respectively, ranking among the highest in China.^2^ Xinjiang’s unique climate, characterized by drought, abundant sunlight, and substantial diurnal temperature variations, facilitates the production of diverse and specialized crop products, such as Hami melons, pomegranates, and nuts. Notably, Xinjiang is the largest cotton and grape production base in China.^3^

Albeit the various favorable conditions for crop production in Xinjiang, it faces multiple challenges hindering agricultural sustainable transformation in the long term. Aridity is the most prominent challenge. Xinjiang’s water resources are unevenly distributed, with some regions experiencing severe water shortages and droughts. These conditions are likely to worsen with global warming, thereby posing a considerable threat to agricultural production.^4^ Another challenge is the increased use of agricultural inputs, including fertilizer and mulching films. Previous research has revealed that alongside increased yield, N fertilizer application rates for cotton production in Xinjiang increased from 150 kg N ha^−1^ in the 1980s to 598 kg N ha^−1^ in 2020, exceeding the national average level.^5^ This has led to the accumulation of N in soil, increasing N surface runoff and leaching. More than 50% of the arable lands in Xinjiang are covered by mulching film, with residuals reaching 255 kg ha^−1^—five times larger than the national average level.^6^

To combat negative resource and environmental conditions and support stable crop production, a series of solutions have been developed and applied since the 1990s, such as drip irrigation under film,^7^ fertigation,^8^ biodegradable film,^9^ and formula fertilizer.^10^ Currently, the film coverage rate of cotton in Xinjiang has reached 100%, and it has the largest area of drip irrigation worldwide. Although studies have demonstrated the positive outcomes of these techniques in improving resource use efficiency and yield, total agricultural inputs have grown annually, particularly inputs of chemical fertilizer and mulching films.^11^ The marginal benefits brought by these agricultural inputs are declining, while the environmental consequences are escalating. Yet, limiting inputs and optimizing management practices on the ground faces multiple barriers, including farmers’ knowledge and acceptance, economic costs, and feasibility. Overall, there are substantial opportunities to improve the sustainability of crop production systems in Xinjiang. This includes the need for ongoing technical support (efficient water usage, optimization of agricultural inputs) and policy support (financial assistance and long-term planning).

Despite having an arid climate and an uneven distribution of water resources, Xinjiang remains China’s one of the most important agricultural regions. Xinjiang represents a typical context for oasis, irrigation, and mechanized agriculture, and its experience in making full use of sunlight and water resources can serve as a model for similar regions worldwide. However, Xinjiang’s agricultural system also faces a series of obstacles in its transformation toward sustainability, the most prominent of which are difficulties in increasing yields, and excessive use of chemical fertilizer and agricultural films, which have restricted local economic growth and environmental protection. These obstacles are also faced by most countries and regions in the world. Taking Xinjiang as an example, this article reviews crop production process in Xinjiang from 2000 to 2020, and analyzes the experiences and problems in the agricultural development process in arid areas. Building on previous research, we proposed policy and technical strategies tailored to the context of Xinjiang, which also has guiding significance for other arid regions worldwide, such as the Middle East, Central Asia, and North Africa.

## Current status of crop production in Xinjiang

### Distribution of crop production area and climatic characteristics

**Figure 1:**
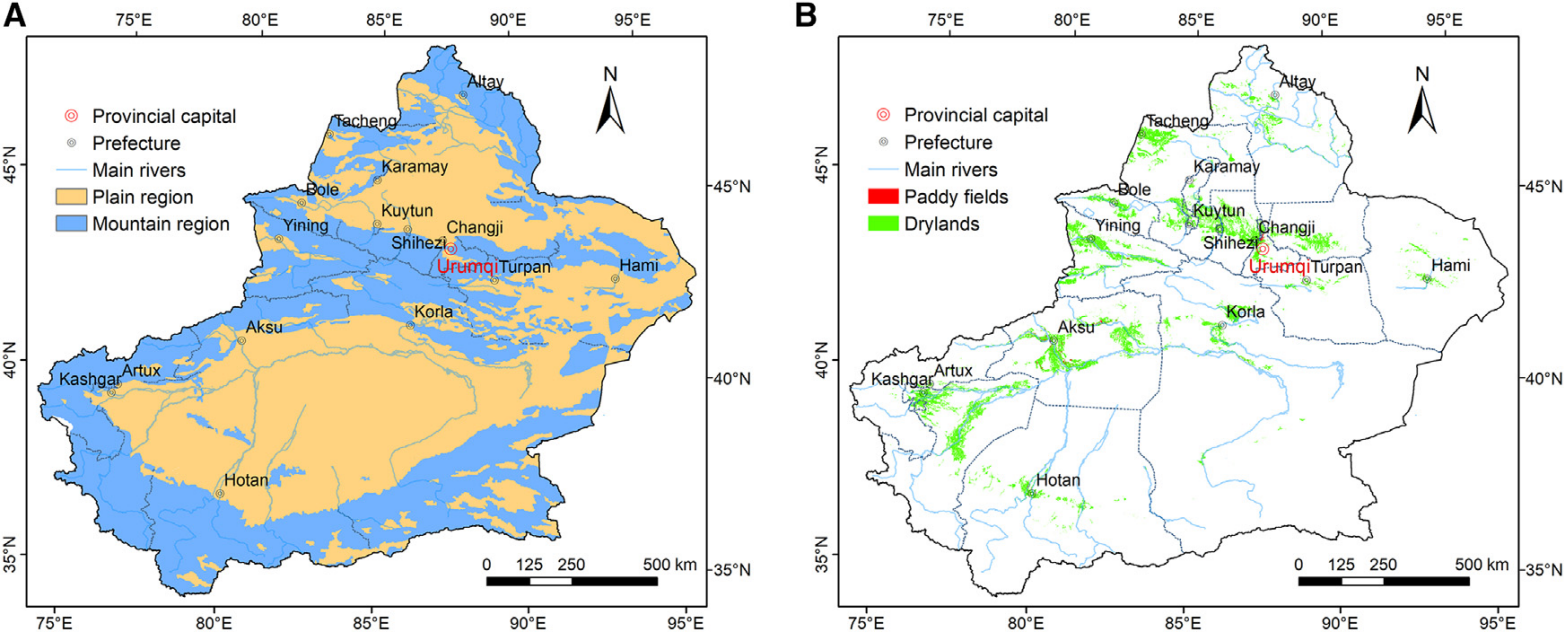
Topographic zoning and distribution of arable lands in Xinjiang (A) Topographic zoning. (B) Distribution of arable lands. The classification criteria in (A) is referenced from Chai et al.^12^ The data used in (B) comprises five periods: 2000, 2005, 2010, 2015, and 2020, serving as the average representation of the spatial distribution of arable lands from 2000 to 2020. Data were derived from Resource and Environmental Science and Data Center (https://www.resdc.cn).^13^

Geographically, the region of Xinjiang is often described as “three mountains with two basins,” referring to the Altai Mountains in the north, the Kunlun Mountains in the south, and the Tian Shan Mountains dividing the northern Junggar Basin (also known as Dzungarian Basin) and the southern Tarim Basin (Figure 1A). Over 99% of the arable lands are drylands, whereas less than 1% of the arable lands are paddy fields. Arable lands are primarily distributed in the western and central regions of Xinjiang, particularly in Kashgar, Aksu, Tacheng, and Changji (Figure 1B).

**Figure 2:**
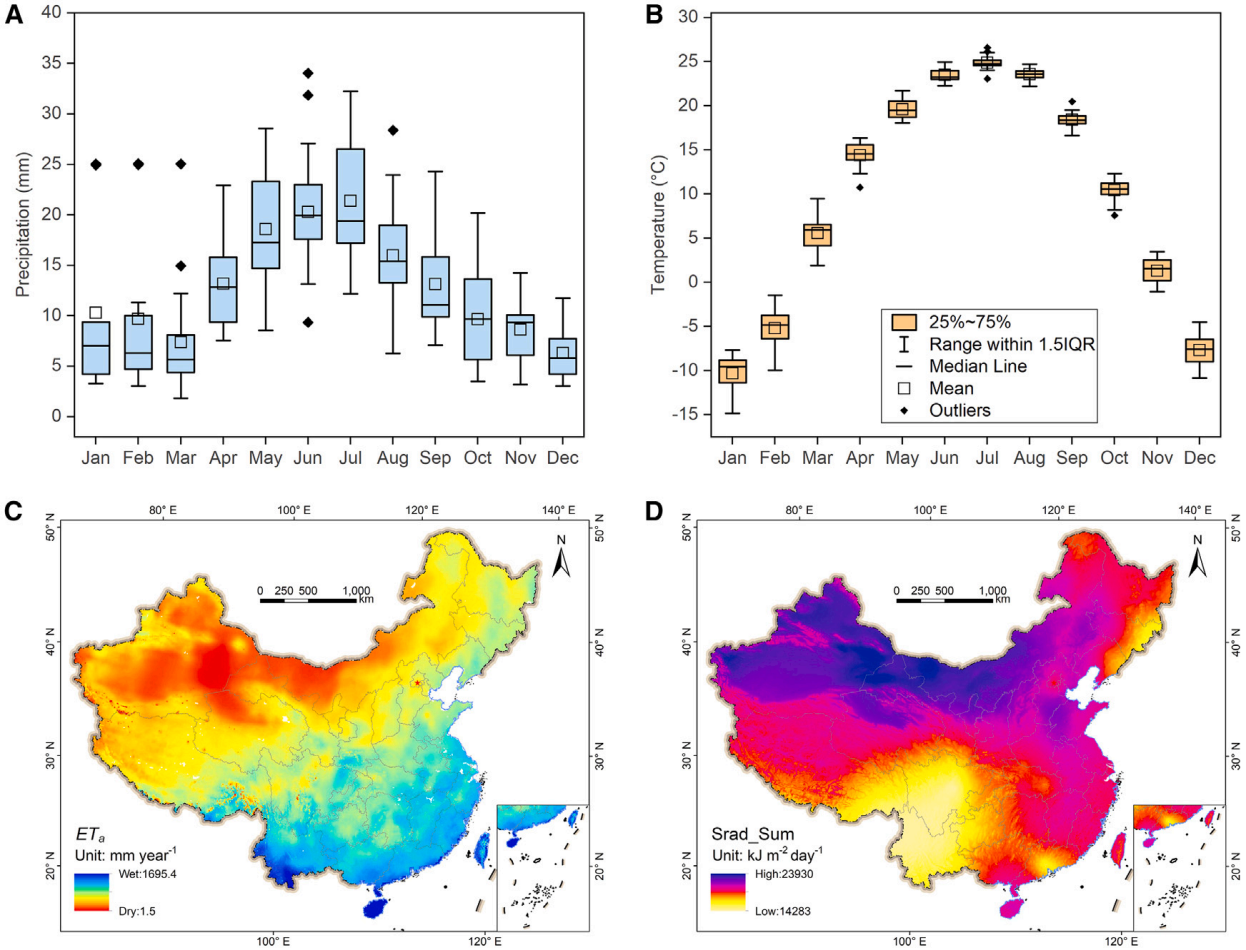
Meteorological characteristics of Xinjiang and China (A) Precipitation of arable lands in Xinjiang; (B) Temperature of arable lands in Xinjiang; (C) Actual evapotranspiration (ET_*a*_) in China; (D) Solar radiation intensity of summer in China. Data in (A) and (B) was derived from Peng et al.^15^; data in (C) and (D) was derived from the World Climate database (https://worldclim.org)^16^ and the National Tibetan Plateau Data Center (http://data.tpdc.ac.cn).^17^^,^^18^

The unique geographic and climatic characteristics of Xinjiang make its agricultural challenges distinct from those in other regions of China. Xinjiang is located far from the ocean, situated deep inland, and surrounded by high mountains, which hinder the arrival of maritime air currents. This geographical setting results in a distinct temperate continental climate. The average annual precipitation for Xinjiang’s arable lands is 155 mm, which is primarily concentrated between April and October (Figure 2A). Peak precipitation occurs in June and July with monthly levels exceeding 20 mm. The average annual temperature is 10°C, but it varies greatly across months. The average monthly temperature reaches its lowest point at −10°C in January and gradually rises to its peak at 25°C in July before gradually decreasing again (Figure 2B). The multi-year average actual evapotranspiration (*ET*_*a*_) map of China shows that the Xinjiang region is more arid than the other areas (Figure 2C). Summer (June, July, and August) is a critical period for crop growth in the Xinjiang region. As illustrated in Figure 2D, the Xinjiang region has greater summer solar radiation (Srad_Sum) intensity than other regions in China. Meanwhile, the average monthly sunshine duration in the Xinjiang region, situated in the northwestern part of China, is 228.0 h, which is considerably higher than that in the southeastern region (including Guangdong, Guangxi, Fujian, etc.), with 150.4 h, and that in the central region (including Anhui, Henan, etc.), with 179.9 h.^14^ Consequently, the pronounced characteristics of the region, including high rates of evapotranspiration, intense solar radiation, and extended daylight hours, have collectively contributed to the development of its unique agricultural attributes.

### Sowing structure variations and major agricultural products

**Figure 3:**
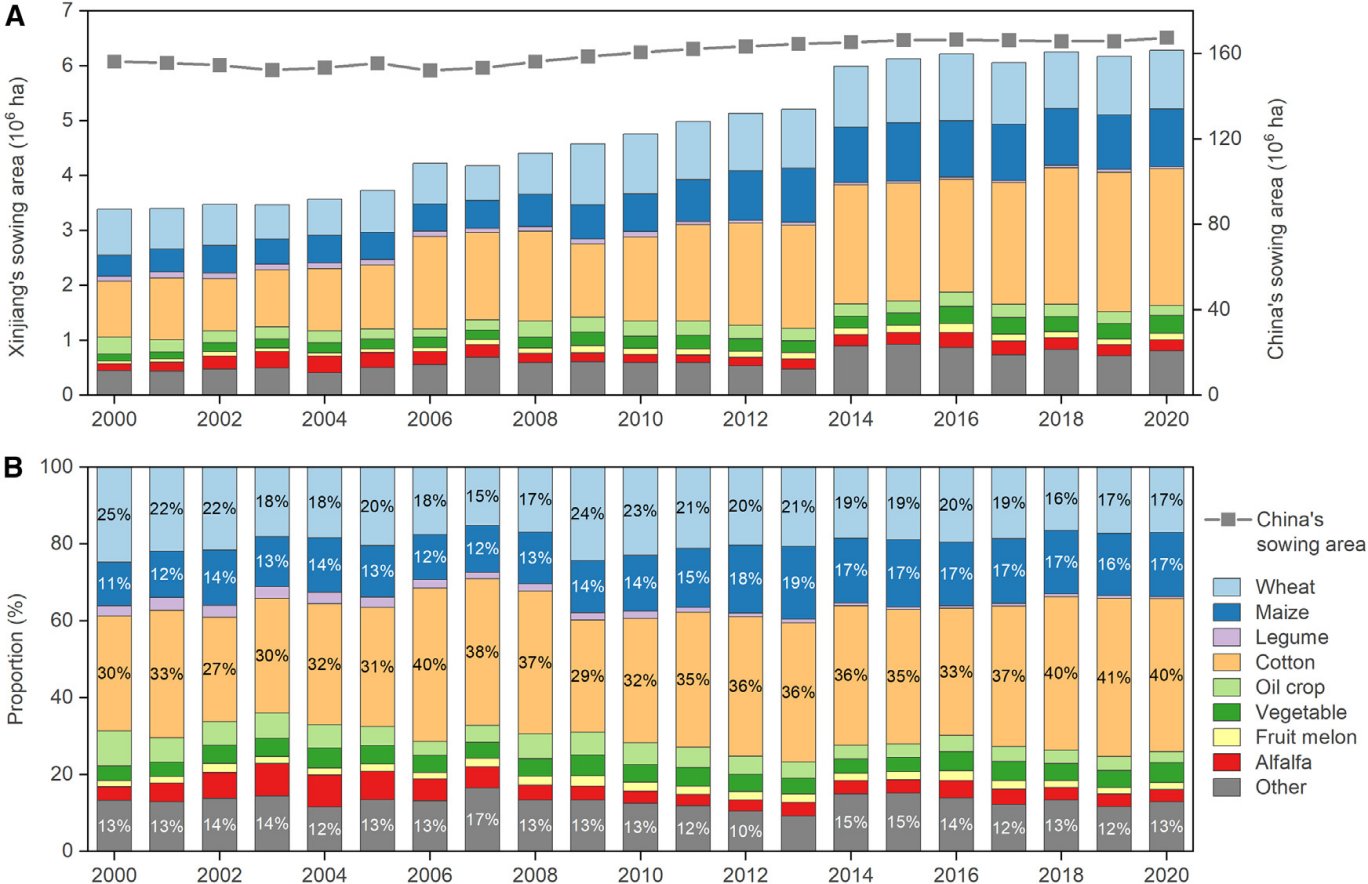
Changes of cropping status in Xinjiang from 2000 to 2020 (A) Sowing area. (B) Cropping pattern. Data were derived from Statistic Bureau of Xinjiang Uygur Autonomous Region^11^ and National Bureau of Statistics of China.^3^

The sowing area in Xinjiang increased from 2000 to 2020, representing 26% of the total increase in sowing area across China during this period (Figure 3A). This indicates that Xinjiang contributes substantially to maintaining the total area of arable land in China. Consequently, more efforts are required to ensure the quality of newly added arable land, enhance soil fertility, and develop sustainable management solutions to safeguard national food security.

Cotton dominates Xinjiang agriculture, comprising 40% of the total sowing area in 2020 (Figure 3B), 90% of China’s production, and 20% of global production.^19^ Along with cotton, wheat, and maize consistently ranked among the top three crops in Xinjiang, showing an upward trend in sowing area from 2000 to 2020, with increases of 147%, 175%, and 27%, respectively (Figure 3A). Meanwhile, oil crops, vegetables, melons, and alfalfa together comprise 13–22% of the sowing area in Xinjiang, with relatively stable sowing areas.

**Figure 4:**
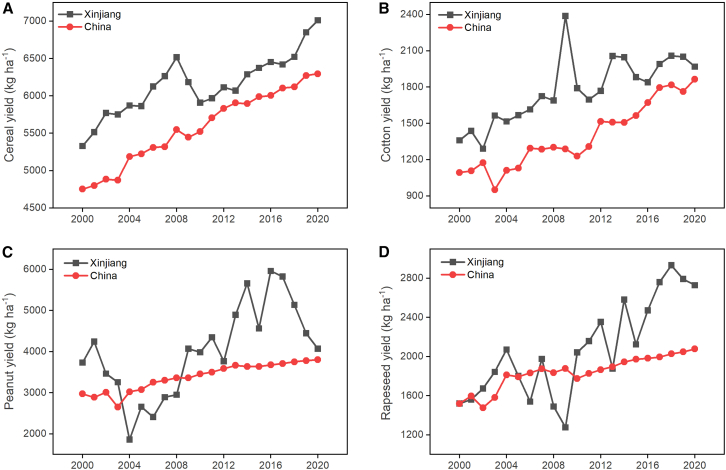
Changes in main crop yield per unit area in Xinjiang compared to the national average from 2000 to 2020 (A) Cereal; (B) Cotton; (C) Peanut; (D) Rapeseed. Data was derived from Statistic Bureau of Xinjiang Uygur Autonomous Region^11^ and National Bureau of Statistics of China.^3^

Xinjiang’s crop yield is higher than the national average. The cereal yield (including wheat, maize, rice, and barley) in Xinjiang is particularly high, consistently reaching 3–18% higher than the national average, with yields reaching 7012 kg ha^−1^ in 2020 (Figure 4A). Similarly, Xinjiang’s cotton yield consistently surpassed the national average between 2000 and 2020, although the difference had declined by 2020 (Figure 4B). Peanut and rapeseed yields in Xinjiang have fluctuated greatly, ranging from 1860 to 5963 kg ha^−1^ and 1276 to 2933 kg ha^−1^, respectively (Figures 4C and 4D). In 2020, Xinjiang’s peanut yield approximated the national average, whereas its rapeseed yield was 31% higher than the national average.

Cotton production in Xinjiang occupies a pivotal strategic position in the region’s economy and development. In 2023, the total cotton production in Xinjiang reached 5.11 million tons, representing 91% of China’s total cotton production and maintaining Xinjiang’s position as the top producer in China for 30 consecutive years. Moreover, Xinjiang’s cotton production surpassed that of the United States, which is the world’s third-largest raw cotton producer, and fulfilled 60% of China’s raw cotton requirements for its textile industry.^20^ In 2021, Xinjiang’s cotton gross product was 121 billion CNY, accounting for 54.2% of Xinjiang’s primary industry (236 billion CNY), and for 7.6% of Xinjiang’s gross product in 2021.^21^^,^^22^ Cotton cultivation has emerged as the principal source of income for farmers in Xinjiang, with primary cotton-producing counties contributing over 60% of farmers’ earnings. Furthermore, the cotton industry chain facilitated the employment of 215 thousand individuals, highlighting the textile and garment industry as an important employment avenue in the region.^21^

Along with cotton production, Xinjiang’s grape farming plays an influential role on the economy. In 2021, Xinjiang’s grape growing area was 21 thousand ha, yielding 3.27 million tons of grapes, which accounted for 21.8% of China’s total grape production^3^^,^^22^; and the grape wine production was 170 thousand kL, which accounted for 24.5% of China’s total grape production, making Xinjiang the largest production base of grape wine in China.^23^ The government has continued to promote the development of the grape industry. According to the Xinjiang government’s development program, a target of 300 thousand kL of grape wine production was set for Xinjiang in 2025, which is expected to stimulate the local tourism industry and promote industrial optimization.^24^^,^^25^ The grape industry has become a characteristic agricultural sector in the Xinjiang region, fostering the diversification of local agriculture.

## Major barriers to transitioning from conventional management to sustainable management

### Water scarcity

**Figure 5:**
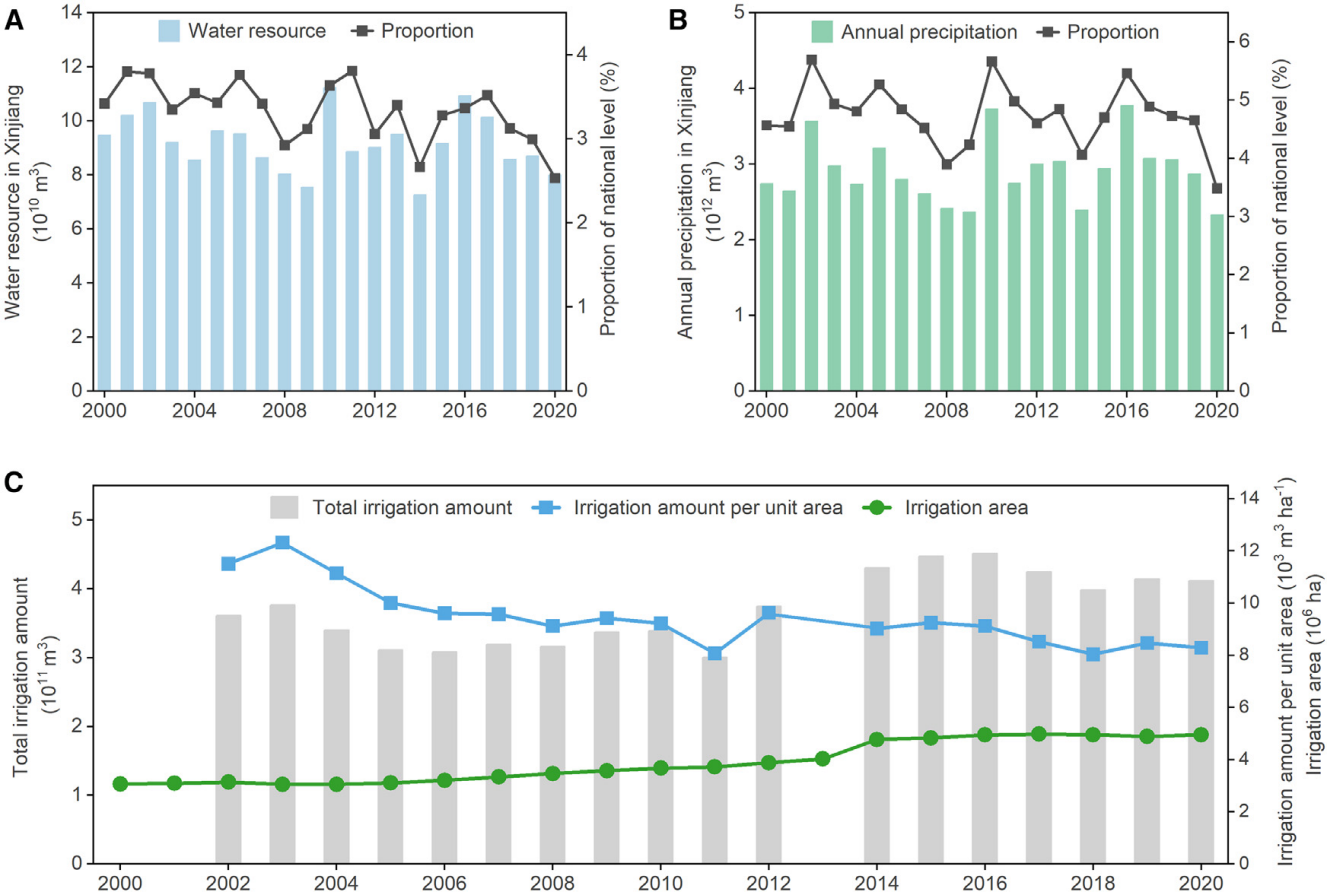
Water resources and irrigation in Xinjiang from 2000 to 2020 (A) Water resources. (B) Annual precipitation. (C) Irrigation amount. Data was derived from Water Resources Department of Xinjiang Uygur Autonomous Region^28^ and Ministry of Water Resources of PRC.^29^

Despite accounting for 17.3% of China’s land area, Xinjiang contains only approximately 3.3% of the country’s average annual water resources, including surface water and groundwater (9.2 × 10^10^ m^3^ in total), and 4.7% of the country’s annual average precipitation (2.9 × 10^11^ m^3^) (Figures 5A and 5B). Therefore, water scarcity is a major concern for this region. The variability within this region is also high. The annual water resources and annual precipitation within Xinjiang have fluctuated, with *coefficients of variation* of 11.5% and 14.2%, respectively. Low soil moisture, longer sunshine durations, and high evapotranspiration rates make many locations within Xinjiang susceptible to drought during years of low precipitation.^26^^,^^27^ For instance, in 2014, Xinjiang experienced its lowest water resources and annual precipitation levels from 2000 to 2020, leading to a drought-damaged area of 3.7 × 10^5^ ha and an area of 4.4 × 10^4^ ha with no harvest.

Irrigation accounts for a large proportion of water utilization in Xinjiang. Between 2000 and 2020, the amount of water irrigated per unit area decreased steadily; however, the expansion of total arable lands led to an increase in the total irrigation volume during this time (Figure 5C). Xinjiang has achieved remarkable progress in the conservation and efficient utilization of water, with water consumption dropping from 12 × 10^3^ m^3^ ha^−1^ in the early 2000s to 8.3 × 10^3^ m^3^ ha^−1^ in 2020. However, the irrigation area in Xinjiang increased from 3.1 × 10^6^ ha in 2000 to 5 × 10^6^ ha in 2020. Consequently, total irrigation volume has continued to increase, highlighting the urgent need to address the conflicts between agricultural water demands and water scarcity.

### Fertilizer overuse

**Figure 6:**
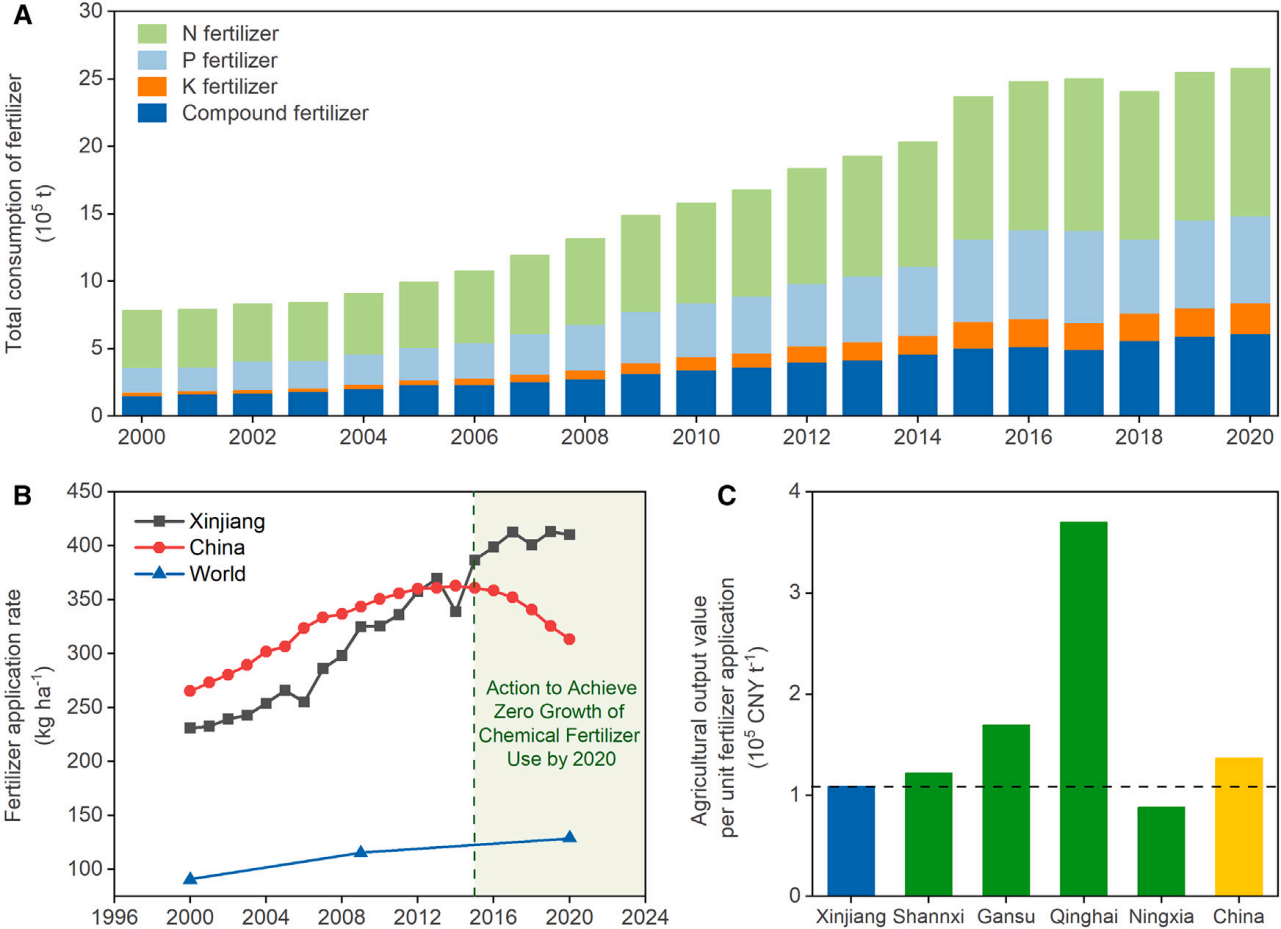
The current status of fertilizer utilization in Xinjiang (A) Total consumption of fertilizer between 2000 and 2020 in Xinjiang; (B) Fertilizer application rate per unit area between 2000 and 2020; (C) Agricultural output value per unit fertilizer application in 2020. Data were derived from Statistic Bureau of Xinjiang Uygur Autonomous Region,^11^ National Bureau of Statistics of China,^3^ and Food and Agriculture Organization of the United Nations.^30^

Along with an increase in Xinjiang’s total irrigation volume, fertilizer use has also increased. The consumption of chemical fertilizer increased by 3.3 times from 2000 to 2020. As the most important fertilizer, N fertilizer usage increased by 158% over these 20 years, while the consumption of other chemical fertilizer increased by even larger increments. In particular, application of P, K, and compound fertilizer increased by 251%, 846%, and 305%, respectively (Figure 6A).

The continuous increase in total fertilizer consumption in Xinjiang can be attributed not only to an expansion in the sowing area but also to an increase in the application rate per unit area (from 231 kg ha^−1^ in 2000 to 410 kg ha^−1^ in 2020; Figure 6B). Although the national average also showed an upward trend in the early years (2000–2014), after the promulgation of the “Action to Achieve Zero Growth of Chemical Fertilizer Use by 2020” measured by the Chinese government in 2015, the national average fertilizer application rate began to decline, reaching 313 kg ha^−1^ in 2020. However, this policy did not effectively reduce the fertilizer application rate in Xinjiang. Xinjiang has exceeded the national average level since 2013, with the disparity widening over time. From a global perspective, China faces a severe issue of excessive fertilizer application, far surpassing the global average. Therefore, there is an urgent need to address fertilizer overuse in both Xinjiang and China as a whole.

The agricultural output value per unit of fertilizer application in Xinjiang was 1.1 × 10^5^ CNY t^−1^, which was below the average level of neighboring provinces (i.e., Shaanxi, Gansu, and Qinghai) and the national level, albeit slightly exceeding that of Ningxia. This indicates that Xinjiang has the potential to reduce fertilizer consumption, improve crop productivity, and enhance the economic benefits of efficient fertilizer utilization.

### Mulching film overuse and residues

**Figure 7:**
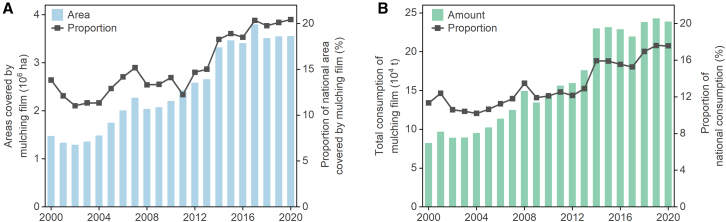
Mulching film utilization in Xinjiang (A) Areas covered by mulching film between 2000 and 2020; (B) Total consumption of agricultural mulching film between 2000 and 2020. Data were derived from Statistic Bureau of Xinjiang Uygur Autonomous Region^11^ and National Bureau of Statistics of China.^3^

Agricultural mulching films have various benefits such as conserving moisture, increasing soil temperature, inhibiting weed growth and pest invasion, and promoting crop growth.^31^^,^^32^ Mulching films have been widely used in Xinjiang for cotton production since being introduced in the region by the Japanese in the 1980s. From 2000 to 2020, the area covered by mulching film in Xinjiang increased from 1.5 × 10^6^ ha to 3.6 × 10^6^ ha; consequently, the total consumption of mulching film grew from 8.2 × 10^4^ t to 2.4 × 10^5^ t during this time (Figure 7). In 2020, Xinjiang accounted for 20% of the national area covered by mulching film and 18% of the national mulching film consumption. Much of this mulching film is used for the cotton production in the region, with a coverage rate of 100%.

The difficulty in recycling these films has led to the growing issue of farmland pollution.^33^ According to a survey conducted by Zhang et al.,^34^ 61% of the observed sites in Xinjiang presented film residue exceeding the national film residue standard (75 kg ha^−1^). The residual film in the soil may weaken or offset the yield-enhancing effects of film application.^35^ Mulching film technology is evolving from “white revolution” to “white pollution,”^36^ posing multiple threats to the sustainable development of fragile oasis agriculture and the local environmental health.^37^

## Strategies for sustainable agriculture in Xinjiang

### Precise irrigation and fertilization

Considering the challenges of an arid climate and limited water supply, Xinjiang has adopted the practice of drip irrigation under film. It was introduced in 1996 by the Xinjiang Production and Construction Corps and was inspired by a technique developed in Israel. Following development of water-saving technologies and facilities, drip irrigation coverage in Xinjiang expanded to a total of 4.28 × 10^6^ ha by the end of 2021. This constitutes 61% of Xinjiang’s cultivated area, making it the largest area in the world that utilizes drip irrigation technology.^38^

Moreover, water-soluble fertilizer can be distributed through the drip lines, a method known as fertigation, which not only improves water and fertilizer use efficiency but also minimizes human labor inputs.^8^ Research was conducted to determine the best combination of irrigation amount and fertilization rate for drip irrigation in Xinjiang. Wang et al.^7^ explored the coupling effects of water and fertilizer on cotton systems in Northern Xinjiang and concluded that an irrigation amount of 362–463 mm ha^−1^ and a fertilizer rate (N:P_2_O_5_:K_2_O) between 213:85:43 and 368:147:74 kg ha^−1^ achieved maximum benefits in terms of cotton yield, economic benefits, and water use efficiency. Wu et al.^8^ conducted a two-year field experiment in northern Xinjiang and found that a 272 kg ha^−1^ N rate combined with 400 mm irrigation resulted in the highest seed cotton yield and N use efficiency compared with other treatments. Ma et al.^39^ conducted field experiments in southern Xinjiang to compare the effects of conventional and reduced N fertilizer applications on cotton systems under a drip irrigation quota of 480 mm ha^−1^. They found that when the N fertilizer application rate decreased from 476 kg N ha^−1^ (conventional management) to 317–395 kg N ha^−1^, the cotton yield increased by 12–21% and N leaching decreased by 20–33%.

In addition to cotton, water-fertilization management of cash crops has also been explored. Li et al.^10^ reported that for grape cultivation in northern Xinjiang, irrigation ranges between 334 and 348 mm and N:P_2_O_5_:K_2_O fertilization ranging from 320:400:880 to 392:490:1077 kg ha^−1^ can simultaneously achieve multiple objectives, such as high grape yield, grape quality, net profit, and water use efficiency. Overall, these findings demonstrate that comprehensive water and fertilizer management strategies in Xinjiang can achieve economic and environmental synergies—enhancing both, without compromising either.

### Organic fertilizer substitution

In addition to precise fertilization, substituting chemical fertilizer with organic fertilizer can effectively curb the overuse of chemical fertilizer in Xinjiang. Currently, organic fertilizer in the region is mostly derived from crop residue, green waste, and livestock manure.^40^ Judicious utilization of organic feedstock in arid areas has been shown to bring various economic and environmental benefits, such as establishing healthy microflora, improving soil quality, and boosting crop production.^41^

The application of organic fertilizer can be divided into two types: directly use on farmlands and conversion into liquids for application through drip irrigation. For direct use, Zhang et al.^42^ found that compared with other treatments, residue incorporation + organic manure and residue incorporation + organic manure + NPK fertilizer showed greater carbon sequestration capability and yield increase in seed cotton. For conversion, both organic and chemical fertilizer were dissolved entirely in the tank and then applied with drip irrigation. Compost tea, a liquid fertilizer derived from diverse organic sources, can be applied using drip irrigation as a partial substitution for organic fertilizer, which has been shown to improve nutrient utilization by cotton and soil quality in Xinjiang.^43^ Chen et al.^44^ conducted a two-year field experiment in eastern Xinjiang to determine the best organic-chemical fertilization strategies for drip-irrigated grapes. They recommended using a 1:1 ratio of organic to chemical fertilizer, combined with an irrigation amount of 720 mm throughout the grape growing cycle. Industrial organic wastewater containing N, P, K, and organic matters can also be converted into liquid organic fertilizer products. For instance, Hao et al.^45^ explored the effects of applying industrial organic wastewater through drip irrigation on cotton production in Xinjiang, and concluded that organic wastewater applied at a rate of 1329 kg ha^−1^ combined with N:P_2_O_5_:K_2_O fertilizer application at a rate of 182:104:76 kg ha^−1^ not only reduced chemical fertilizer use, but also increased yield and chemical fertilizer use efficiency.

In summary, organic fertilizer substitution is a sustainable way to dispose of organic waste and alleviate environmental burdens. Simultaneously, it facilitates the reuse of nutrients, enhances crop production, and reduces reliance on chemical fertilizer. Nevertheless, there is a huge potential to explore border feedstocks for organic fertilizer production and develop tailored products that can facilitate the wider utilization of organic fertilizer throughout Xinjiang.

### Mulching film management

Despite the benefits of mulching film utilization, residual film has become a prominent issue, causing a series of negative consequences, such as damaging soil structure,^46^ inhibiting root development,^47^ and reducing nutrient supply to crops.^48^ The quantity of residual mulching film in soils is affected by various factors, such as the film utilization amount, film thickness, mulching years, recovery method, crop type, etc.^34^ To address the problems associated with residual mulching film, solutions such as applying thicker mulching film and developing environmentally friendly mulching film should be explored.

Film thickness is a key factor affecting the recovery rate of mulching films; the thicker the film, the easier it is to recover after use.^36^ According to agriculture plastics and environment Europe, the residual rates of mulching films with thicknesses of 0.010, 0.020, and 0.025 mm are 68%, 25%, and 10% by mass, respectively.^49^ Polyethylene is the most widely used material in mulching film production in China. According to the national standard for “Polyethylene Blown Mulch Film for Agricultural Uses (no. GB 13735-1992)” promulgated in 1992, the thickness of mulching film must be 0.008–0.020 mm, allowing an error of +/− 0.003 mm. Consequently, farmers tend to purchase thinner films to reduce costs, incentivizing film production companies to produce ultra-thin films with a thickness of 0.006–0.008 mm. In contrast, the common thickness of mulching film in Europe falls between 0.010 and 0.025 mm, and that in Japan falls between 0.020 and 0.030 mm, ensuring that the film is relatively intact after use and leading to a lower residual rate after mechanized recovery.^50^ In 2017, China released a new national standard for mulching film manufacturing (no. GB 13735-2017), which increased the required thickness to 0.010–0.030 mm. However, this standard is yet to be fully applied in Xinjiang.

In addition to enhancing film thickness, developing and applying environmentally friendly films is an alternative solution that can save labor and costs for recovering and disposing of residual waste. Biodegradable film and photodegradable films are research hotspots and have been widely applied in field trials.^51^ Two types of polymer blends have been used for biodegradable films: natural polymer blends (e.g., starch, cellulose, and chitosan) and synthetic polymer blends (e.g., polylactic acid, polybutylene adipate terephthalate [PBAT], and polycaprolactone).^52^ At the end of their life cycles, biodegradable films can be transformed into H_2_O, CO_2_, CH_4_, and biomass by native soil microorganisms.^31^ Light-sensitizing additives have been combined with petroleum-based ingredients to produce photodegradable films. Wang et al.^53^ tested four biodegradable films in cotton fields in northern Xinjiang and found that the PBAT film performed similarly to the polyethylene film in terms of improving the cotton yield. Moreover, as the PBAT film had the lowest residual waste, it was recommended as an alternative to conventional polyethylene film.

However, several studies have shown that the photodegradable films may undergo either premature or delayed degradation, posing unfavorable conditions for agricultural production.^54^ The degradable effects of photodegradable films are also unpredictable because light-sensitizing additives cause the physical breakdown of films without altering their chemical properties.^51^

### Suitability evaluations of arable lands and crops

Evaluations the suitability of arable lands and crops provides another means of facilitating the efficient use of arable land resources. Although Xinjiang has experienced intense agricultural development, this development has not always been substantiated by thorough evaluations. Such an evaluation can determine the suitability of uncultivated land for conversion to arable lands, thereby expanding arable land resources.^55^ Alternatively, an evaluation can also be used to identify the optimal zones for cultivating various crops, aiming to improve the existing cropping structure, pattern, and resource use efficiency.^56^ For instance, based on the Penman-Monteith model of evapotranspiration, cotton in Xinjiang requires the most water during the mid-growth period from July to August, with an average value of 394 mm for the entire region,^57^ whereas winter wheat requires the most water during the mid-growth period around May, with an average value of 258 mm for the entire region.^58^ By strategically aligning the spatial distribution of crops, staggered water usage for different crops can be implemented to achieve efficient water utilization, thereby promoting the conservation and efficient use of water resources and avoiding waste.

In 2021, the Chinese government promulgated the “National Technical Program for the Survey and Evaluation of Arable Land Reserve Resources,” proposing a series of indicators for evaluating the cultivation potential of land.^59^ This program mandates systematic evaluation, requiring a comprehensive consideration of factors such as ecology, climate, soil, and location, with the aim of screening arable land reserve resources. Xinjiang covers a vast area with complex climatic, topographic, soil, hydrological, and geological conditions, all of which play a crucial role in shaping land use and influencing crop growth.^27^ Hence, it is necessary to undertake the suitability evaluation of arable lands and crops in Xinjiang, aiming to evaluate the suitability of arable land and crops in Xinjiang to exploit the largest potential for developing efficient production of staple crops, cotton, and melons.

Assessing the suitability of land in Xinjiang for farming can be achieved through analysis of data on the characteristics of climate, soil, hydrology, topography, geology, and human activities in land currently designated as arable. Species distribution models (SDMs) are a promising approach that is widely used to evaluate crop suitability.^56^^,^^60^ They derive the potential extent of a species from species distribution information and relevant environmental factors.^61^^,^^62^ Many SDMs have been developed to predict species, including the maximum entropy model, enetic algorithm for Ruleset prediction, ecological niche analysis and so forth.^63^^,^^64^^,^^65^ Overall, utilizing SDMs to analyze suitable crop planting areas while considering the current distribution of arable lands enables the formulation of scientific recommendations for optimizing the planting structure in various regions according to production requirements in Xinjiang.

### Stakeholder participation and policy support

From crop production to resource use efficiency and environmental sustainability, these outcomes interrelated in positive or negative ways, thereby generating synergies or trade-offs.^66^ Multi-objective coordination requires the engagement of various stakeholders in decision-making processes, which is the linchpin toward achieving sustainable agriculture.^67^ Multi-stakeholder participation generally involves efforts from national or local governments, legislative bodies, external funding sources, research institutions or universities, agricultural input manufacturing factories, and farmers.^68^

Currently, various innovative collaboration platforms have been developed worldwide, serving for pinpointing bottlenecks in sustainable transformation and facilitating negotiations among diverse stakeholders. For instance, Schut et al.^69^ designed the rapid appraisal of agricultural innovation systems (RAAIS), focusing on analyzing different dimensions of problems (such as biophysical, technological, socio-cultural, economic, institutional, and political), different levels (national, regional, and local), and constraints and interactions among various stakeholders (farmers, government, researchers, etc.). This approach involves gathering representatives from different stakeholders and holding workshops to identify the primary limiting factors within their respective domains. Through the RAAIS, the most urgent problems in a particular area can be diagnosed, thereby determining priority areas for resolution. Science and technology ackyards (STB) hubs provide another model, initiated by China agricultural university, to empower smallholder farmers through collaboration with scientists, agricultural inputs production companies, local agricultural service organizations, and leading farmers. Zhang et al.^70^ found that through the STB platform, the average wheat yield of 71 leading farmers in Quzhou County (a typical grain-producing county in the North China Plain) increased from 68% of potential yield to 97% over five years, while the county’s five-year average yield increased from 63% of potential yield to 80%. In 2019, the Xinjiang Kunyu Jujube STB was officially established, serving the jujube industry in Kunyu City. This STB focuses on labor-saving and efficient cultivation, technology transfer from scientists to farmers, and adjustment of the local jujube industry structure, which is dedicated to developing a sustainable development model of high-quality products, environmentally friendliness, and increased income for farmers.

Although various innovative management strategies and suitable arable areas have been proposed, farmers’ adoption of sustainable practices requires concrete incentives, such as subsidies, qualified products, and machinery services.^71^ The government plays a crucial role in organizing all stakeholders through coercive or incentive-based approaches. In the past 20 years, a series of government actions have been undertaken to support sustainable crop production in Xinjiang, mainly focusing on water conservation, fertilization optimization, mulching film utilization and recovery, and mechanization promotion (Table 1).

**Table 1:** Key actions to support sustainable crop production in Xinjiang since 2000

Category	No.	Title	Key contents	Issuer (Year)
Water-saving	1	Emergency Teleconferencing for Drought Relief in Xinjiang	a. Provide a subsidy of 100 CNY mu^−1^ for farmlands with high-tech water-saving irrigation. b. Provide fiscal subsidy of 100,000 CNY for each new drought-resistant well drilled in Northern Xinjiang.	XJ Gov (2008)^72^
	2	Xinjiang Turpan Water Conservation Project	a. Provide a loan of $100 million to assist in water resources management in Turpan Basin. b. Reduce the risk of flooding and groundwater overdraft, increase water productivity.	World Bank (2010)^73^
	3	“13^th^ Five-Year Plan” for Adding 100 Million Mu of High-Efficiency Water-Saving Irrigation Area	a. Add 100 million mu of high-efficiency water-saving irrigation areas nationwide over five years (2016–2020). b. Allocate 20 million mu of high-efficiency water-saving irrigation areas among provinces annually.	PRC Gov (2017)^74^
	4	Water Saving Action Implementation Plan in Xinjiang	a. Determine water-saving goals for 2020, 2022, and 2025. b. Strengthen organization leadership, improve fiscal and tax policies, expand financing models.	XJ Gov (2019)^75^
	5	“14^th^ Five-Year Plan” for Building a Water-Saving Society in Xinjiang	a. Add 3.45 million mu of high-efficiency water-saving irrigation areas over five years (2021–2025). b. Increase irrigation water use efficiency to 0.585.	XJ Gov (2023)^76^
Fertilization	6	Formula Fertilization Based on Soil Testing Pilot Program	a. Carry out soil testing and formula fertilization pilot projects across the country. b. Provide subsidies for important steps in soil testing and formula fertilization. c. Provide technical training for farmers on soil testing and formula fertilization.	PRC Gov (2005)^77^
	7	Platform on Characteristic Forest and Fruit Resource Investigation and Soil Testing and Fertilization Technology	a. Conduct a survey of orchard soil and fertility conditions in Xinjiang. b. Propose fertilizer strategies according to regions, fruit tree species, and growth stages.	XJ Gov (2012)^78^
	8	Action to Achieve Zero Growth of Chemical Fertilizer Use by 2020	a. Achieve zero growth in use of chemical fertilizer for major crops by 2020. b. Improve fertilizer composition, organic fertilizer utilization, formula fertilizer, and application method.	PRC Gov (2015)^79^
	9	Notice on Issuance of the Results of “2020 Xinjiang Soil Testing and Formula Fertilization Certification Companies”	a. 34 companies were identified as soil testing and formula fertilization certified companies in 2020. b. Certified companies must produce high-quality fertilizer in accordance with projects.	XJ Gov (2020)^80^
	10	Notice on Strengthening the Production of Organic Fertilizer in Xinjiang	a. Achieve the application of no less than 58 million tons of organic fertilizer in Xinjiang in 2020. b. Reduce excessive use of chemical fertilizer, and produce qualified and efficient organic fertilizer.	XJ Gov (2020)^81^
	11	Guidance on Scientific Fertilization of Major Crops in Spring 2022	a. Apply fertilizer according to target yield, crop, soil, and soil testing result. b. Use organic fertilizer to partially replace chemical fertilizer.	XJ Gov (2022)^82^
Mulching film	12	Measures to Further Strengthen the Management of Pilot Projects for Scientific Use and Recycling of Mulching Film	a. Producers must add corporate logos on films to facilitate traceability and supervision. b. Users need to establish records of product use and recycling, and keep them for at least two years.	XJ Gov (2022)^83^
	13	Specification for Classification and Gradation of Waste Plastic Mulch Film	a. Specifies the requirements for inspection rules, classification methods, and grading methods for waste mulch films	XJ Gov (2023)^84^
	14	Regulations on Agricultural Mulching Film Management in Xinjiang	a. Clarify the obligations and legal responsibilities of producers, sellers and users. b. Determine technical standards for mulch film recycling and processing companies.	XJ PC (2024)^85^
Mechanization	15	Agricultural Mechanization Promotion Law	a. Support farmers and organizations to use advanced and applicable agricultural machinery. b. Forbid compelling farmers or organizations to purchase designated agricultural machinery products.	PRC PC (2004)^86^
	16	Regulations on Promotion of Agricultural Mechanization in Xinjiang	a. Support the research and development of new machinery, ensure the quality of machinery. b. Support socialized services, provide financial support.	XJ PC (2017)^87^
	17	Opinions on Accelerating the Development of Mechanization in Facility Farming	a. Promote the mechanization of production operations and intelligent facilities and equipment. b. Develop innovative models of social services.	PRC Gov (2020)^88^
	18	Guiding Opinions on the Implementation of Agricultural Machinery Renewal Subsidies	a. Provide appropriate subsidies to individuals and organizations for scrapping old agricultural machinery. b. Promote advanced, efficient, environmentally friendly, and energy-saving machinery.	PRC Gov (2020)^89^
	19	Guidance on Optimizing Cotton Variety Structure and Improving Full-process Mechanization Capabilities	a. Improve comprehensive mechanization rate to over 90% by 2025. b. Improve agricultural machinery purchase subsidy policy.	XJ Gov (2020)^90^

Mu, unit of area, corresponding to 1/15 of a hectare, or about 667 m^2^. XJ Gov: The People’s Government of Xinjiang Uygur Autonomous Region of China; PRC Gov: The Government of the People’s Republic of China; XJ PC: The People’s Congress of Xinjiang Uygur Autonomous Region; PRC PC: The National People’s Congress of the People’s Republic of China.

Policy support can be roughly divided into four categories: (1) national or local plans and programs, (2) laws and regulations, (3) technical guidance, and (4) financial support. National or local plans and programs typically set macro-level objectives that delineate the directions and specific tasks of agricultural development. As part of the “13th Five-Year Plan (2016–2020),” the Chinese government set the goal of adding 100 million mu of high efficiency water-saving irrigation area nationally. In 2015, the Ministry of Agriculture and Rural Affairs of PRC proposed the “Action to Achieve Zero Growth of Chemical Fertilizer Use by 2020” to curb the excessive use of chemical fertilizer.

Underpinning these plans and programs, laws and regulations serve as mandatory measures to maintain social order, protect the rights of individuals and groups, and establish responsibilities. For instance, China introduced the “Agricultural Mechanization Promotion Law” in 2004 to standardize the quality of agricultural machinery products while also protecting farmers’ rights and fostering technological innovation. In 2016, Xinjiang implemented the “Regulations on Agricultural Mulching Film Management in Xinjiang” to oversee the production, sale, and use of mulching films and formulate punishment measures for companies producing mulching films that fail to meet the specified standards.

Technical guidance aims to provide specific scientific recommendations for farmers to achieve income increases and efficient resource use. For example, the “Guidance on Scientific Fertilization of Major Crops in Spring 2022,” initiated by the Department of Agriculture and Rural Affairs of Xinjiang, provides explicit instructions on fertilizer rates, formulas, and application methods tailored for various major crops, such as wheat, maize, cotton, and tomatoes. Financial support typically takes the form of subsidies, loans, and research funding to bolster infrastructure construction, technology adoption, and scientific innovations associated with sustainable agriculture. This includes initiatives such as building drought-resistant wells and supporting research on new agricultural machinery. Together, all four of these types of policy support can help transform conventional agriculture into sustainable agriculture.

## Conclusion

The arid climate and unique topography of Xinjiang make it the most important region for cotton and grape production in China. From 2000 to 2020, the change in planting patterns occurred mainly in cotton, with a 147% increase in planting area. Xinjiang’s great achievements in agriculture are attributed to its high level of mechanization and vigorous development of precise irrigation and fertilization. Along with the rapid development of Xinjiang’s agriculture, the excessive use of chemical fertilizer and mulching films has caused inefficient resource utilization and increased environmental burdens. To address the conflicts between agricultural development and resource conservation, a comprehensive approach involving technological innovations and assessments of the suitability of arable lands and crops must be taken. Innovative practices can be designed and tested on different crops to identify the best practice that can achieve multiple benefits. Implementing these judicious management strategies also requires the establishment of stakeholder participation platforms, accompanied by supportive programs, regulations, guidance, and funding from the government. Overall, to achieve sustainable agriculture in Xinjiang, both technological effectiveness and farmers’ interests should be considered. In this way, technology can be widely adopted, thereby improving crop yield and quality, increasing farmers’ incomes, enhancing resource utilization efficiency, and reducing environmental impacts. These solutions extend beyond regional borders and offer valuable insights for other regions across the globe predominantly engaged in arid or oasis agriculture.

## Acknowledgments

This work was supported by the Science and Technology Innovation Foundation of the Comprehensive Survey & Command Center for Natural Resources, 10.13039/501100004613China Geological Survey (no. KC20230003), the project of 10.13039/501100004613China Geological Survey (no. DD20242769), the 10.13039/501100001809National Natural Science Foundation of China (no. 42377462), the National Natural Science Foundation of China Youth Fund (no. 42307576), and the Third Xinjiang Scientific Expedition Program (no. 2022xjkk090405).

## Author contributions

Conceptualization: X.Z. and X.G.; data curation: H.L.; writing-original draft: X.Z. and X.G.; writing-review and editing: A.L.Z.; validation: X.L. and J.L; funding acquisition: J.L.

## Declaration of interests

The authors declare no competing interests.

## References

[1] The People's Government of Xinjiang Uygur Autonomous Region of China Overview of Xinjiang Uygur Autonomous Region. https://www.xinjiang.gov.cn/xinjiang/dmxj/dmxj.shtml.

[2] The State Council of PRC Xinjiang's autumn grain harvest passes 90%-Comprehensive mechanization of crop cultivation, planting and harvesting in the region is expected to reach more than 90%. https://www.gov.cn/lianbo/difang/202411/content_6985981.htm.

[3] National Bureau of Statistics of China (2022). China Statistical Yearbook. https://www.stats.gov.cn/sj/ndsj/2022/indexch.htm.

[4] Zhang Q., Li J., Singh V.P., Bai Y. (2012). SPI-based evaluation of drought events in Xinjiang, China. Nat. Hazards.

[5] Lv N., Zhu H., Cheng W. (2022). Feasibility study on reduction of agricultural chemical fertilizer and substitution of bio-fertilizer: An empirical study of cotton survey data in Xinjiang (in Chinese with English abstract). Geogr. Res..

[6] Xu Y., Fang S., Ma X., Zhu Q. (2018). Prevention and control strategy for the pollution of agricultural plastic film (in Chinese with English abstract). Strateg. Stud. CAE.

[7] Wang H., Wu L., Cheng M., Fan J., Zhang F., Zou Y., Chau H.W., Gao Z., Wang X. (2018). Coupling effects of water and fertilizer on yield, water and fertilizer use efficiency of drip-fertigated cotton in northern Xinjiang, China. Field Crops Res..

[8] Wu B., Yang P., Zuo W., Zhang W. (2023). Optimizing water and nitrogen management can enhance nitrogen heterogeneity and stimulate root foraging. Field Crops Res..

[9] Deng L., Yu Y., Zhang H., Wang Q., Yu R. (2019). The effects of biodegradable mulch film on the growth, yield, and water use efficiency of cotton and maize in an arid region. Sustainability.

[10] Li X., Liu H., Li J., He X., Gong P., Lin E., Li K., Li L., Binley A. (2020). Experimental study and multi-objective optimization for drip irrigation of grapes in arid areas of northwest China. Agric. Water Manag..

[11] Statistic Bureau of Xinjiang Uygur Autonomous Region (2021). Xinjiang Statistical Yearbook. https://tjj.xinjiang.gov.cn/tjj/zhhvgh/list_nj1.shtml.

[12] Chai H., Zhou C., Chen X., Cheng W., Ou Y., Yuan Y. (2008). The new methodology of geomorphologic zonalization in Xinjiang based on geographical grid (in Chinese with English abstract). Geogr. Res..

[13] Xu X., Liu J., Zhang S., Li R., Yan X., Wu S. (2020).

[14] Dong G. (2024).

[15] Peng S., Ding Y., Liu W., Li Z. (2019). 1 km monthly temperature and precipitation dataset for China from 1901 to 2017. Earth Syst. Sci. Data.

[16] Fick S.E., Hijmans R.J. (2017). WorldClim 2: new 1-km spatial resolution climate surfaces for global land areas. Int. J. Climatol..

[17] Ma N., Szilagyi J., Zhang Y., Liu W. (2019). Complementary-relationship-based modeling of terrestrial evapotranspiration across China during 1982-2012: Validations and spatiotemporal analyses. JGR Atmos.

[18] Ma N., Szilagyi J. (2019). The CR of evaporation: A calibration-free diagnostic and benchmarking tool for large-scale terrestrial evapotranspiration modeling. Water Resour. Res..

[19] Ministry of Agriculture and Rural Affairs of PRC The "addition and subtraction" behind the high-quality development of Xinjiang's cotton industry. http://www.moa.gov.cn/xw/qg/202311/t20231127_6441336.htm.

[20] The People's Government of Xinjiang Uygur Autonomous Region of China (2023). Overview of modern agricultural development in Xinjiang. https://www.xinjiang.gov.cn/xinjiang/bmdt/202402/97a465946fd94ecca0375577676a2818.shtml.

[21] Xinjiang Uygur Autonomous Region Development and Reform Commission Implementing the spirit of the 20th CPC national congress: High-quality cotton and textile and garment industry cluster in Xinjiang. https://xjdrc.xinjiang.gov.cn/xjfgw/hgjj/202301/f51add2fca264dc2ae8d34c183e8f361.shtml.

[22] Statistic Bureau of Xinjiang Uygur Autonomous Region (2021). Xinjiang Uygur Autonomous Region national economic and social development statistics bulletin. https://tjj.xinjiang.gov.cn/tjj/tjgn/202203/7ab304445f174a7eb1f5165be4f94041.shtml.

[23] Xinjiang Uygur Autonomous Region Development and Reform Commission Research on the development of wine industry in Xinjiang. https://xjdrc.xinjiang.gov.cn/xjfgw/hgjj/202301/8ac1880b0f374bd2bc234ac9404137b5.shtml.

[24] Department of Industry and Information Technology, Xinjiang Uygur Autonomous Region Xinjiang Uygur Autonomous Region wine industry development plan (2018-2025). https://gxt.xinjiang.gov.cn/gxt/gfxwj/201905/45c4f8f7b4d64fa096562f6df76d1b8a.shtml.

[25] Department of Industry and Information Technology, Xinjiang Uygur Autonomous Region Policy interpretation - Xinjiang Uygur Autonomous Region wine industry development plan (2018-2025). https://gxt.xinjiang.gov.cn/gxt/jdhy/202005/f33607c0c87c4331806fdf054e68327a.shtml.

[26] Cao X., Bao Y., Li Y., Li J., Wu M. (2023). Unravelling the effects of crop blue, green and grey virtual water flows on regional agricultural water footprint and scarcity. Agric. Water Manag..

[27] Yao J., Chen Y., Guan X., Zhao Y., Chen J., Mao W. (2022). Recent climate and hydrological changes in a mountain–basin system in Xinjiang, China. Earth Sci. Rev..

[28] Water Resources Department of Xinjiang Uygur Autonomous Region (2020). Water resources bulletin. https://slt.xinjiang.gov.cn/xjslt/c114491/202305/407588b681854bf6b01f63725441f9b6.shtml.

[29] Ministry of Water Resources of PRC (2020). Water resources bulletin of China. http://www.mwr.gov.cn/sj/tjgb/szygb/202107/t20210709_1528208.html.

[30] Food and Agriculture Organization of the United Nations (2022). World food and agriculture- statistical yearbook.

[31] Kasirajan S., Ngouajio M. (2012). Polyethylene and biodegradable mulches for agricultural applications. Agron. Sustain. Dev..

[32] Sintim H.Y., Bandopadhyay S., English M.E., Bary A.I., DeBruyn J.M., Schaeffer S.M., Miles C.A., Reganold J.P., Flury M. (2019). Impacts of biodegradable plastic mulches on soil health. Agric. Ecosyst. Environ..

[33] Yu Y., Zhang Z., Zhang Y., Jia H., Li Y., Yao H. (2023). Abundances of agricultural microplastics and their contribution to the soil organic carbon pool in plastic film mulching fields of Xinjiang, China. Chemosphere.

[34] Zhang D., Liu H.b., Hu W.l., Qin X.h., Ma X.w., Yan C.r., Wang H.y. (2016). The status and distribution characteristics of residual mulching film in Xinjiang, China. J. Integr. Agric..

[35] Yang L., Heng T., He X., Yang G., Zhao L., Li Y., Xu Y. (2023). Spatial-temporal distribution and accumulation characteristics of residual plastic film in cotton fields in arid oasis area and the effects on soil salt transport and crop growth. Soil Tillage Res..

[36] Liu E.K., He W.Q., Yan C.R. (2014). 'White revolution' to 'white pollution'-agricultural plastic film mulch in China. Environ. Res. Lett..

[37] Harms I.K., Diekötter T., Troegel S., Lenz M. (2021). Amount, distribution and composition of large microplastics in typical agricultural soils in Northern Germany. Sci. Total Environ..

[38] Liang F., Li Y., Guan X., Liu H., Yin F. (2023). Progress of effects of long-term field drip irrigation on water and salt balance of farmland in Xinjiang (in Chinese with English abstract). J. Huazhong Agr. Uni..

[39] Ma H., Yang Z., Pu S., Wu X., Wang Z., Ma X., Yang T. (2023). Effect of nitrogen application on nitrogen leaching pattern and leaching loss from drip irrigated cotton fields in Southern Xinjiang. Environ. Res. Commun..

[40] Xu H., Huang X., Zhong T., Chen Z., Yu J. (2014). Chinese land policies and farmers' adoption of organic fertilizer for saline soils. Land Use Policy.

[41] Tao R., Hu B., Chu G. (2020). Impacts of organic fertilization with a drip irrigation system on bacterial and fungal communities in cotton field. Agric. Syst..

[42] Zhang P.p., Xu S.z., Zhang G.j., Pu X.z., Wang J., Zhang W. (2019). Carbon cycle in response to residue management and fertilizer application in a cotton field in arid Northwest China. J. Integr. Agric..

[43] Luo T., Ma L., Wei C., Li J. (2022). Effects of compost tea on the spatial distribution of soil nutrients and growth of cotton under different fertilization strategies. J. Plant Nutr..

[44] Chen R., Chang H., Wang Z., Lin H. (2023). Determining organic-inorganic fertilizer application threshold to maximize the yield and quality of drip-irrigated grapes in an extremely arid area of Xinjiang, China. Agr. Water Manag..

[45] Hao X., Shi X., Khan A., Li N., Shi F., Li J., Tian Y., Han P., Wang J., Luo H. (2022). Industrial organic wastewater through drip irrigation to reduce chemical fertilizer input and increase use efficiency by promoting N and P absorption of cotton in arid areas. Agriculture.

[46] Yan C., Liu E., Shu F., Liu Q., Liu S., He W. (2014). Review of agricultural plastic mulching and its residual pollution and prevention measures in China (in Chinese with English abstract). J. Agric. Resour. Environ..

[47] Hu Q., Li X., Gonçalves J.M., Shi H., Tian T., Chen N. (2020). Effects of residual plastic-film mulch on field corn growth and productivity. Sci. Total Environ..

[48] Ibarra-Jiménez L., Lira-Saldivar R., Valdez-Aguilar L., Lozano-Del R. (2011). Colored plastic mulches affect soil temperature and tuber production of potato. Acta Agr. Scan. Soil Plant Sci..

[49] Xu Y. (2023). Series Report of Sino-European Sustainable Transition Towards Circular Economy.

[50] Xie X., Xu H., Zhang W., Zhao M. (2023). What government interventions are effective in regulating the use and recycling of high-standard mulch film in China?. Environ. Sci. Pollut. Res. Int..

[51] Xue Y., Sun Z., Ju X., Xi B., Jin T., Jia T. (2020). Current status of research and application od degradable materials for agricultural soil films (in Chinese with English abstract). China Plast..

[52] Akhir M.A.M., Mustapha M. (2022). Formulation of biodegradable plastic mulch film for agriculture crop protection: A review. Polym. Rev..

[53] Wang Z., Wu Q., Fan B., Zhang J., Li W., Zheng X., Lin H., Guo L. (2019). Testing biodegradable films as alternatives to plastic films in enhancing cotton (*Gossypium hirsutum* L.) yield under mulched drip irrigation. Soil Tillage Res..

[54] Greer L., Dole J.M. (2003). Aluminum foil, aluminium-painted, plastic, and degradable mulches increase yields and decrease insect-vectored viral diseases of vegetables. HortTechnology.

[55] Akpoti K., Kabo-bah A.T., Zwart S.J. (2019). Review - Agricultural land suitability analysis: State-of-the-art and outlooks for integration of climate change analysis. Agric. Syst..

[56] Li X., Wu K., Hao S., Yue Z., Ran Z., Ma J. (2023). Mapping cropland suitability in China using optimized MaxEnt model. Field Crops Res..

[57] Wang M., Yang Q., Zheng J., Liu Z. (2016). Spatial and temporal distribution of water requirement of cotton in Xinjiang from 1963 to 2012 (in Chinese with English abstract). Acta Ecol. Sin..

[58] Xuan J., Zheng J., Liu Z. (2015). Spatiotemporal characteristics of water requirement of wheat as influenced by climate in Xinjiang in recent 50 years (in Chinese with English abstract). Res. Soil Water Conserv..

[59] Ministry of Natural Resources of PRC Notice on carrying out the survey and evaluation of national cultivated land reserve resources. https://www.gov.cn/zhengce/zhengceku/2021-07/10/content_5624029.htm.

[60] Thibaud E., Petitpierre B., Broennimann O., Davison A.C., Guisan A. (2014). Measuring the relative effect of factors affecting species distribution model predictions. Methods Ecol. Evol..

[61] Guisan A., Thuiller W. (2005). Predicting species distribution: Offering more than simple habitat models. Ecol. Lett..

[62] Zhao X., Lei M., Wei C., Guo X. (2022). Assessing the suitable regions and the key factors for three Cd-accumulating plants (*Sedum alfredii, Phytolacca americana*, and *Hylotelephium spectabile*) in China using MaxEnt model. Sci. Total Environ..

[63] Hengl T., Sierdsema H., Radović A., Dilo A. (2009). Spatial prediction of species’ distributions from occurrence-only records: Combining point pattern analysis, ENFA and regression-kriging. Ecol. Model..

[64] Phillips S.J., Anderson R.P., Dudík M., Schapire R.E., Blair M.E. (2017). Opening the black box: an open-source release of Maxent. Ecography.

[65] Townsend Peterson A., Papeş M., Eaton M. (2007). Transferability and model evaluation in ecological niche modeling: a comparison of GARP and Maxent. Ecography.

[66] Balmford A., Amano T., Bartlett H., Chadwick D., Collins A., Edwards D., Field R., Garnsworthy P., Green R., Smith P. (2018). The environmental costs and benefits of high-yield farming. Nat. Sustain..

[67] Lacoste M., Cook S., McNee M., Gale D., Ingram J., Bellon-Maurel V., MacMillan T., Sylvester-Bradley R., Kindred D., Bramley R. (2022). On-farm experimentation to transform global agriculture. Nat. Food.

[68] Guo X.X., Li K.L., Liu Y.Z., Zhuang M.H., Wang C. (2022). Toward the economic-environmental sustainability of smallholder farming systems through judicious management strategies and optimized planting structures. Renew. Sustain. Energy Rev..

[69] Schut M., Klerkx L., Rodenburg J., Kayeke J., Hinnou L.C., Raboanarielina C.M., Adegbola P.Y., van Ast A., Bastiaans L. (2015). RAAIS: Rapid appraisal of agricultural innovation systems (Part I). A diagnostic tool for integrated analysis of complex problems and innovation capacity. Agric. Syst..

[70] Zhang W., Cao G., Li X., Zhang H., Wang C., Liu Q., Chen X., Cui Z., Shen J., Jiang R. (2016). Closing yield gaps in China by empowering smallholder farmers. Nature.

[71] Piñeiro V., Arias J., Dürr J., Elverdin P., Ibáñez A.M., Kinengyere A., Opazo C.M., Owoo N., Page J.R., Prager S.D., Torero M. (2020). A scoping review on incentives for adoption of sustainable agricultural practices and their outcomes. Nat. Sustain..

[72] The People's Government of Xinjiang Uygur Autonomous Region of China High-tech water-saving irrigation farmland subsidy of $100 per acre. https://www.xinjiang.gov.cn/xinjiang/xjyw/200807/a27e6133d229479a8745a04538f74e9f.shtml.

[73] World Bank Group World Bank loan to support Xinjiang Turpan Qanerjing conservation and water-saving irrigation project. https://www.shihang.org/zh/news/press-release/2010/06/17/world-bank-support-water-conservation-protect-ancient-well-systems-chinas-xinjiang#.

[74] The State Council of PRC Five departments jointly issued the "13th Five-Year Plan" to add 100 million mu of highly efficient water-saving irrigation area implementation programme. https://www.gov.cn/xinwen/2017-02/27/content_5171293.htm.

[75] The People's Government of Xinjiang Uygur Autonomous Region of China Xinjiang Uygur Autonomous Region water conservation action implementation programme. https://www.xinjiang.gov.cn/xinjiang/bmdt/202001/c1bcf00b022f42c0bb5bc2a769ddec4b.shtml.

[76] Xinjiang Uygur Autonomous Region Department of Water Resources Xinjiang Uygur Autonomous Region water conservation society construction "14th Five-Year Plan" released and implemented. https://slt.xinjiang.gov.cn/xjslt/c114425/202301/f6c2b5ef05844e638ec10ba7ee58b9fe.shtml.

[77] Ministry of Agriculture and Rural Affairs of the People's Republic of China Notice on effectively carrying out the pilot work on soil testing and formulated fertilization in 2005. https://www.moa.gov.cn/nybgb/2005/dsyq/201806/t20180618_6152543.htm.

[78] Xinjiang Academy of Forest Compilation of results of research projects 2012-2023 (I). http://xjlky.cn/web/details?typeid=12&id=3057.

[79] Ministry of Agriculture and Rural Affairs of the People's Republic of China Notice of the general office of the ministry of agriculture on printing and distributing the “Implementation Plan for Promoting the Action Plan for Zero Growth in Fertilizer Use by 2020”. http://www.zzys.moa.gov.cn/gzdt/201505/t20150525_6309954.htm.

[80] Department of Agriculture and Rural Affairs of Xinjiang Notice on the issuance of the results of the "2020 Xinjiang soil testing and formulation fertilization recognition enterprises". https://nynct.xinjiang.gov.cn/xjnynct/c113635/202004/fa35dc877e91413285460a4aa547b2ae.shtml.

[81] Department of Agriculture and Rural Affairs of Xinjiang Notice on strengthening the accumulation of organic fertilizers in the Autonomous Communities. https://nynct.xinjiang.gov.cn/xjnynct/c113635/202004/faebb769bdc242ec89f8fd05904e0071.shtml.

[82] Department of Agriculture and Rural Affairs of Xinjiang (2022). Guidance on scientific fertilization of major crops in spring. https://nynct.xinjiang.gov.cn/xjnynct/c113605/202203/df719fd3b59344eba92feada83c926fb.shtml.

[83] Department of Agriculture and Rural Affairs of Xinjiang Measures to further strengthen the management of the pilot project on scientific use and recycling of mulch film. https://nynct.xinjiang.gov.cn/xjnynct/c113595/202410/8dedf6ed544248c6911fc30161aab86f.shtml.

[84] Xinjiang Uygur Autonomous Region Market Supervision and Administration Bureau Consultation on the proposed publication of local standards for the classification and grading of waste mulch. https://scjgj.xinjiang.gov.cn/xjaic/c112980/202307/848f5688ef244391a5b41f799a7878a5.shtml.

[85] Postal Administration of Xinjiang Uygur Autonomous Region Regulations on the management of agricultural mulch in Xinjiang Uygur Autonomous Region. https://xj.spb.gov.cn/xjyzglj/c100065/c100066/202406/b8336214032b4f6d952491f24feb670c.shtml.

[86] The State Council of PRC Law of the People's Republic of China on the promotion of agricultural mechanization. https://www.gov.cn/zhengce/2005-06/27/content_2602161.htm.

[87] Xinjiang Agricultural and Rural Mechanization Development Center Regulations on the promotion of agricultural mechanization in Xinjiang Uygur Autonomous Region. https://www.xjnj.gov.cn/xjnj/zhengce/202204/5670bc7f48824a189200aa0f51f5ab58.shtml.

[88] The State Council of PRC Opinions of the Ministry of Agriculture and Rural Affairs on accelerating the development of facility planting mechanization. https://www.gov.cn/zhengce/zhengceku/2020-06/30/content_5522757.htm.

[89] National Development and Reform Commission of PRC Guidance on the implementation of subsidies for the scrapping and renewal of agricultural machinery. https://www.ndrc.gov.cn/xwdt/ztzl/tddgmsbgxhxfpyjhx/gzdt/202404/t20240417_1365733.html.

[90] The People's Government of Xinjiang Uygur Autonomous Region of China Guiding opinions on optimizing cotton variety structure and enhancing full-process mechanization capability. https://www.xinjiang.gov.cn/xinjiang/gfxwj1/202311/2809120755ea4a70bbd75c910fba5d1b.shtml.

